# What is cultural in cultural commons?

**DOI:** 10.12688/openreseurope.20908.1

**Published:** 2026-02-17

**Authors:** Lyudmila Petrova, Marilena Vecco, Daniele Tammaro, Arjo Klamer

**Affiliations:** 1 Erasmus University Rotterdam Erasmus School of History Culture and Communication, Rotterdam, The Netherlands; 2 Burgundy Business School, Dijon, France; 3 University of Pisa, Pisa, Italy; 4 Free University, Amsterdam, The Netherlands

**Keywords:** cultural and social values, cultural commons, cultural goods, shared practices

## Abstract

David Throsby made significant contributions to cultural economics, making the field visible in academic economics. His work extended beyond standard economic tools, recognizing that artistic practices require new economic approaches. Building on Throsby’s value approach, this paper explores how cultural goods differ from standard economic products, emphasizing their shared and common nature. It examines the cultural and social dimensions of these goods, analysing their role within the commons. By integrating diverse literature sources, the study proposes a taxonomy of commoning practices, addressing research gaps and future inquiries. It highlights the multiplicity of values—such as aesthetic, social, and historical—demonstrating how cultural goods function beyond financial worth within communities and shared practices.

**JEL Classification** Z11

## Introduction

David Throsby has made important contributions to the practices of cultural economics. His survey article in the Journal of Economic Literature ( Throsby, 1994) made the field visible in the community of academic economics. In a wide range of articles he showed how the economic way of thinking sheds light on the labour market of artists, cultural heritage, diversity and subsidies to the arts. His presidential address to the association of Cultural Economists loaded Bourdieu’s notion of cultural capital for cultural economics ( Throsby, 1999). His books on cultural economics ( Throsby, 2001) and cultural policy ( Throsby, 2010) became highly cited standard works.

David Throsby went beyond the conventional application of economic methodologies to the arts. He did not just bring in the market logic, the role of prices and rational choice to make sense of artistic practices, but he was also among the first cultural economists who realized that the study of artistic practices generates insights that require adjustments in, and additions to, the standard toolbox of economists. Although always modest, Throsby called upon fellow economists outside the cultural field to pay attention; they may stand to learn from the artistic world.

He discovered, for example, that artists and other creatives are not primarily focused on the realization of financial values (such as price, income), but used the notion of values in ways that are unknown to most economists. He found and named a series of these values: aesthetic, spiritual, social, historical, symbolic, authenticity and educational values ( Throsby, 2001). He also constructed and frequently used the model of the cultural industries, which distinguishes various cultural activities in separate circles, with the creative arts constituting the core ( Throsby, 2008).

With these insights, the field opened up for approaches that deviate, and sometimes are in contradiction with, the standard economic approach. In this contribution, we follow through on Throsby’s value - based approach (we coined the term ourselves). We do so by readdressing the notion of “goods” to demonstrate that cultural goods are different from standard economic goods and products, as they are goods with the values that Throsby identified. We will extend the analysis by introducing the notion of shared and common goods and common practices to assert that an important characteristic of the arts is that they involve shared and common practices, and that their values need to be shared. Next, this paper explores both cultural and social dimensions of cultural goods that function as a commons. More specifically, it aims first to analyse the relationships between cultural goods and the commons, focusing on the cultural and social values that these goods yield in commons-related contexts. Second, by integrating different literature sources, this paper explores the commons in the arts and culture in order to build a conceptual framework which addresses research gaps and paths of further inquiry in the above-mentioned relationships. The ambitious goal of this study is to propose a taxonomy of commoning practice in respect to the multiplicity of values and functions of cultural goods.

From the cultural economics perspective, cultural goods exist as a result of human creativity and yield cultural values while being aesthetically appealing or/and intellectually inspiring; in addition, they also feature intellectual properties ( Throsby, 2001). Their production is characterized by a high level of uncertainty ( Caves, 2000) because of the idiosyncratic nature of cultural goods ( Santagata, 2006). An important part of the discussion of cultural goods is the values that they can yield, which is a focus of study for cultural economists (e.g. Ginsburgh, 2003; Hutter & Shusterman, 2006; Hutter & Throsby, 2008; Klamer, 2002; Klamer, 2017; Snowball, 2011; Throsby, 2001; Vecco & Imperiale, 2017). Beyond economic value, cultural economists distinguish between cultural and social values ( Klamer, 2003; Throsby, 1999).

In her seminal work, *Governing the Commons: The Evolution of Institutions for Collective Action* (1990), Ostrom was the first scholar to introduce the concept of the commons and highlight its economic implications. From the commons perspective, a cultural good is interpreted as a common or a shared good, that is, one which is based on shared practices or the commons ( Bertacchini *et al.*, 2012; Klamer, 2017). Here, the constitutive characteristics of the community become important for understanding the social dynamics within which cultural goods function. Common goods are those that are non-excludable; another characteristic of theirs is that their value increases with the intensity of usage ( Bertacchini *et al.*, 2012).

While the commons perspective prioritizes the realization of social values such as belonging, identity, social distinction, freedom, solidarity, trust, tolerance and responsibility, the cultural economic perspective emphasises that, to fulfil its function, a cultural good needs to achieve both cultural, i.e. artistic/aesthetic/intellectual/symbolic and social values. Cultural goods yield artistic and social merits only when people share and use them. Here, the assumption is that the realisation of, that is, make real these social and cultural values of a cultural good depends on the different capacities of individuals and communities to deal with these values, and to achieve them within various contexts. We argue that cultural values and social values within cultural commons practices are intertwined yet distinct concepts, and they can motivate different outcomes.

Building on the concept of constructed commons ( Madison *et al.*, 2010) and the understanding that cultural commons should go beyond simply understanding their structure and governance, we propose examining the reasons why people participate and contribute to cultural commons. The present analysis will address these differences by adresing what social dynamics qua diverse relationships underpin the cultural practices. In other words, comprising both types of culture, the common practices might be informed not only by artistic values, but also by the specific culture that shapes the traditions of a community and its norms of behaviour. The tensions between those two sets of values and corresponding practices usually produce relational dynamics which can affect the governance structures, and the way the cultural commons acts upon their purposes.

To understand how these differences work, we integrate literature related to (cultural) commons, cultural goods and values and apply bibliometric analysis to examine the intersection of cultural and social values in relation to the nature of the shared cultural resources, and propose a taxonomy of cultural commons–orientated practices.

## Commons and the practice of commoning

Recently, both management and economics researchers have shown a growing interest in the terminology and practices associated with commons.

### Commons

The concept of the commons has undergone significant evolution in organizational and management scholarship. Rose (2020) traces this trajectory from Hardin’s early framing of scarcity and overuse to Ostrom’s institutional solutions, underscoring how the commons is now understood as a dynamic governance system rather than a fixed resource.

Research supports this by identifying various theories, constructs, and practices related to commons-based organizational designs. This body of work examines individuals collaborating for the common good and developing collective forms of production, distribution, management, and ownership of common goods ( Hess, 2008; Ostrom, 1990). As Bollier (2007) notes, this trend reflects the emergence of a broader “commons paradigm.”

The idea of the commons is historically grounded in the long-standing practices of human societies to jointly manage and govern shared resources ( Bravo & De Moor, 2008; Meyer, 2020).

Scholars have increasingly observed a conceptual shift in the understanding of the commons -from naturally shared resources that exist independently (such as communal land or forests) to collectively created and managed human-made resources aimed at addressing shared needs (including open-source software, renewable energy production, and complementary currencies) ( Dardot & Laval, 2014; Meyer, 2020; O'Mahony & Ferraro, 2007). In the entrepreneurship literature, extensive research on collective and community enterprises has highlighted the importance of community engagement ( Haugh, 2007), and values and ideology ( Dey, 2016; Fournier, 2013) in establishing commons.

Ostrom’s (1990) seminal research examined common-pool resources, which are rival and costly to exclude, such as forests or irrigation systems. These must be distinguished from public goods, which are likewise non-excludable but do not face rivalry in consumption. Contemporary literature points to a shift in the commons concept: moving from naturally occurring shared resources to intentionally created resources, including digital platforms, renewable energy projects, and alternative financial systems ( Meyer, 2020). A lot of attention has been paid to urban commons (Feinberg *et al.*, 2021), while the concept of cultural commons remains mainly underexplored.

Beyond resource allocation, scholars stress that commons should be seen as value practices. Pazaitis *et al.* (2022) develop a ‘theory of value as a commons,’ highlighting that digital platforms and collaborative production generate shared value that cannot be reduced to traditional market or state mechanisms. Commons regimes are also inherently dynamic. Nayak & Berkes (2022) emphasises that they emerge, evolve, and sometimes collapse depending on shifting balances of cooperation and contestation, reinforcing the importance of studying commons as processes rather than static arrangements. Critical voices remind us that commons governance can fail if theoretical assumptions are applied too rigidly. Saunders (2014) argues that oversimplified applications of common-pool resource theory risk undermining the very initiatives they are meant to support, suggesting that context-sensitive approaches are vital.

### Commoning

The idea of “commoning” refers to the collective practice of generating and governing resources together. It emphasises horizontal collaboration, innovation in organisational forms, and cooperative efforts to produce sustainable and shared outcomes ( Bollier & Helfrich, 2015; De Angelis, 2017). De Angelis ( 2017, p. 10) conceptualizes the commons as a *“social system”* consisting of three interrelated dimensions: *(1)* a shared pool of tangible and intangible resources; *(2)* a community that together uses, produces, reproduces, and distributes these resources; and *(3)* the practice of *commoning*, understood as form of collaboration. The term *commoning* refers to the collective and often innovative organizational practices through which communities create, manage, and govern shared resources. These practices involve new configurations of labor and responsibilities that enable governance based on principles of collective action established by the community itself. As De Angelis ( 2017, p. 30) further explains, commoning entails generating *“use value for a plurality,”* thereby constituting a community that *“claims and sustains the ownership of the common good”* through *“relational values.”.* Commoning facilitates collective forms of resource governance and ownership directed toward the common good, as seen in cooperatives, community-based enterprises, and peer-production initiatives. It embodies a strong experimental dimension, characterized by *“joint action”* to co-create solutions, products, and services through cooperative practices that advance shared goals and long-term sustainability ( Bollier & Helfrich, 2015, p. 1). Through such practices, commons foster the enduring resilience and adaptive capacity of community self-organization.

## Cultural goods and shared values and practices: a commons perspective

Cultural commons can be defined as “the individual or collective cultural productions, expressions and practices marked by a form of inclusion and following participatory governance” ( de Clippele, 2023, p. 4). In other words, individuals or collectives can be said to be part of cultural commons by virtue of participating in, and contributing to, common practices. Consequently, ownership of a commons is commonly held, and ordinarily unsupported by legal contracts or laws. A common practice can occur within a physical space, such as a gallery or, alternatively, it can be something that people can do within a variety of settings; for example, a certain artistic practice can take place wherever the participants choose to do their thing ( Bertacchini *et al.*, 2012).

### Cultural practices

For the purpose of the analysis presented here, we adopt a recent conceptualisation of cultural practices from UNESCO (2025):

“Cultural practices encompass all activities undertaken by artists, performers, groups, communities and audiences within the CCE. These activities are oriented towards the creation of cultural and creative products, the sharing of knowledge, or the appreciation of unique symbolic values conveyed through the CCI [cultural and creative industries] and cultural and natural heritage” (p.19).

Three different categories derive from this definition: artistic practices related to cultural and creative practitioners; living heritage practices related to cultural groups and communities, and practices of cultural participation by audiences. Artistic practices describe the activities undertaken by artists and creatives, and considers the production of art/culture-related goods. The activities undertaken by groups and communities are called ‘living heritage’ practices and represent the values, traditions, beliefs, knowledge and skills of those communities that are “identifying, safeguarding, protecting and transmitting their cultural and natural heritage” ( UNESCO, 2025, p. 20). These practices form part of communities’ daily lives. Cultural participation practices are understood as being transversal, insofar as the audience can be passive (using, observing, buying cultural goods) and/or active (co-creating, organising, producing cultural goods) participants in the generation of cultural values.

In order to understand these practices, we propose studying them through the lens of cultural economics. The cultural economics perspective of cultural commons involves understanding cultural resources as cultural goods, which are shared and managed collectively (thus also shared and common goods), and which serve to create an impact through the realisation of different values. A central dimension of the discourse concerning this relationship pertains to the forms of value generated by cultural goods (see Klamer, 1996; Klamer, 2002; Throsby, 2001). These contributions are situated within the valuation approach tradition that characterizes scholarship in cultural economics ( Dekker, 2014).

From the perspective of cultural economics, cultural goods are the result of human creativity and yield cultural values, whilst simultaneously being aesthetically appealing and/or intellectually inspiring, and containing intellectual properties ( Throsby, 2001). Moreover, their production is characterized by high levels of uncertainty ( Caves, 2000) due to their idiosyncratic nature ( Santagata, 2006). Acknowledging on the one hand the multidimensionality of cultural values, and on the other the importance of communities of realising these values, we find it is important to understand how both sets of values are realised when cultural goods function within a commons context. If a community is focused on the cultural dimensions of a good, its purpose should be to preserve, enhance and affirm cultural value via its social and cultural practices. Here, cultural practices are defined as sense-making activities or achievements in the arts and cultural sectors, which are related to symbolic, aesthetic, historic and intellectual meanings. These practices generate, for example, ideas and content that artists create and share with others. Social practices here relate to the broader, anthropological sense of culture qua values, norms and behaviour.

From the perspective of commons, a cultural good can be said to be a common or a shared good; that is, one which is based on shared practices or the commons ( Bertacchini *et al.*, 2012; Klamer, 2017). Here, the constitutive characteristics of the community are of critical relevance to understanding the social dynamics within which cultural goods function. Common goods are those that are non-excludable, which is to say that everyone can enjoy them, and whose value increases the more that they are used ( Bertacchini *et al.*, 2012). In this way, they are important for the collective benefits they produce. They differ from public goods in the sense that they cease to exist when nobody uses them, participates in them or contributes towards them. This is why the common/shared practice is critical. When a common space is abandoned, then the common practice that rendered that space valuable ceases to exist. Therefore, the critical issue with respect to commons is their governance ( Ostrom, 1990).

Cultural heritage and assets might also be perceived as cultural commons, on the premise that they belong to everyone collectively, even if there is no legal ownership ( Harvey, 2011; Klamer, 2017; Pratt, 2009). These can include objects, buildings, artefacts and so on (i.e. tangible things), or practices such as knowledge, language, traditions and so forth (intangible things), as well as cultural landscapes (natural things) ( Throsby, 1999; Vecco, 2010). In this context, cultural commons may be understood as resources produced by previous generations and transmitted to both present and future ones. Although such assets typically lack formal collective ownership, they are nonetheless characterized by a sense of community and shared identity ( Dameri & Moggi, 2021).

### Values and cultural commons

An important aspect of the discussion of cultural goods concerns the use value and non-use value that they can yield (e.g., Ginsburgh, 2003; Hutter & Throsby, 2008; Klamer, 2002, Klamer, 2017; Snowball, 2011; Throsby, 2001). Whilst cultural economists extensively study the use value of the cultural goods which derive from the physical, cultural (buildings, collections, artefacts etc.) and human capital, they also recognize and pay particular attention to the significance of the non-use or non-market value which cultural goods generate, such as cultural enrichment, social cohesion and identity formation. Although these values are not easily quantifiable in monetary terms, they nevertheless contribute towards the overall well-being of society ( Klamer, 2017; Throsby, 2001). The non-use value that cultural goods yield through the flows of cultural services can be broken down into cultural and social non-use values, where “cultural” connotes/refers to the artistic, aesthetic, symbolic and historic functions of the cultural goods. Contrastingly, “social” represents anthropological dimensions in which different norms, values and practices are linked to certain group or community identities ( Klamer, 2003; Petrova, 2020; Throsby, 1999).

While the commons perspective focuses on achieving social values, the cultural economics approach highlights the need for cultural goods to generate multiple forms of value, including cultural (artistic, aesthetic, intellectual, symbolic), social and, when possible, economic value. In many ways, cultural goods attain their artistic and social significance only when they are shared and actively used by people. Ordinarily, cultural goods possess cultural content that is valuable, and therefore should be shared amongst people. For example, a private museum’s artworks have a cultural value that transcends the museum’s legal ownership. Here, the assumption is that the realisation of the social and cultural values of a cultural good occurs in different practices or experiences and, moreover, it is dependent on the capacities of individuals and communities that engage in these practices. They can thus be said to achieve financial value when people are willing to pay to participate in them (for example, when they pay to gain access to an exhibition or a heritage site).

If a community, or a particular group of people such as a collective of artists, strives to realise cultural values, then it must strike the requisite balance between cultural, social and financial values. In order to function as a community, social values such as certain norms, a sense of belonging, mutual trust, identity and so on are important, but they can also come into conflict with those practices geared towards the realisation of artistic values. Cultural practices are activities that take place within the arts and cultural sectors, which are related to symbolic, aesthetic, historic or intellectual meanings. These practices reflect, for example, new ideas and content that artists create and share with others. The experiences of artistic collectives serve to illustrate how artists frequently clash with each other in these practices which, in turn, creates tensions in their social practices.

Bearing in mind that cultural commons share a variety of values that reflect their nature as collective resources (both tangible and intangible) that intend to benefit communities and society, the following analysis aims to address the social dynamics, in the form of diverse relationships that underpin the cultural practices of the cultural commons. Doing so can contribute towards a better understanding of the way a commons performs.

## Methodology

Through a systematic literature review complemented by bibliometric analysis, this paper investigates how cultural and social values intersect in shaping the shared cultural resources - both assets and practices - of organizations oriented toward the cultural commons.

### Systematic literature review and biometric analysis

To consolidate insights across the relevant research domains, we employed a systematic literature review (SLR) following the protocol established by Tranfield *et al.* (2003). In the fields of economics, management, and entrepreneurship, the SLR has become a *“standard method”* ( Denyer & Tranfield, 2009, p. 673) for identifying, analysing, and synthesizing existing scientific evidence in a transparent and replicable manner. This approach is particularly appropriate for emerging or fragmented areas of inquiry, where conceptual boundaries and theoretical frameworks remain underdeveloped ( Denyer & Neely, 2004; Denyer & Tranfield, 2009; Tranfield *et al.*, 2003). Furthermore, the design of SLRs can be adapted to the objectives and scope of specific projects, allowing methodological flexibility while maintaining systematic rigor ( MacPherson & Holt, 2007; Pittaway *et al.*, 2004; Thorpe *et al.*, 2005).

As well as this, we employed bibliometric analysis, a quantitative method for evaluating scholarly literature using statistical and computational techniques. This approach facilitates the systematic examination of academic outputs in order to identify intellectual structures, research trends and emerging gaps or themes in a given field ( van Eck & Waltman, 2014; Zupic & Čater, 2015).

The bibliometric analysis was conducted using the Web of Science (WoS) database, selected for its rigorous indexing standards, comprehensive multidisciplinary coverage and high reliability as a primary source for bibliometric research ( Mongeon & Paul-Hus, 2016). In order to construct the dataset, we performed an initial search using the TOPIC (TS) search option. This option retrieves documents based on selected keywords appearing in titles, abstracts, author keywords and WoS-provided Keywords Plus, ensuring a comprehensive coverage of relevant terms and concepts within the literature. Specifically, we used the following search string: TS=("Cultural common$" OR "Knowledge common$" OR "Digital common$") AND TS=(Cultur*). This query was designed to include contributions related to commons and culture by employing truncation operators (“$” and “*”) to include variations of the search terms. The search was conducted on January 31 ^st^ 2025 without any publication date restrictions to allow for a broad temporal perspective. Additionally, only documents in English were included in the analysis.

The initial search comprised a total of 144 contributions. We then applied a two-step selection process. First, we conducted a title and abstract screening to exclude contributions considered irrelevant or out of scope, resulting in a refined sample of 32 contributions. Next, to ensure comprehensive coverage, the initial database search was supplemented through manual screening and snowballing techniques, which identified additional relevant articles and journals not captured by the automated query ( Denyer & Tranfield, 2009; Pittaway *et al*., 2004). All retrieved records were subjected to a systematic screening process, in which titles, keywords, and abstracts were reviewed for relevance against the inclusion criteria. The corresponding bibliographic metadata were extracted and stored in a dedicated dataset to facilitate traceability and subsequent analysis. We added 12 further contributions identified as relevant to the bibliometric analysis. They were selected by applying the same key words as described above. This process resulted in a final sample of 44 contributions, which formed the basis of our bibliometric analysis ( Petrova *et al.*, 2025).

Finally, to perform the bibliometric analysis presented in the results section, we used Bibliometrix, an open-source package within the R statistical programming environment. Bibliometrix is widely recognized for its robust analytical tools, making it a powerful instrument for conducting bibliometric, scientometric and other types of quantitative analyses ( Aria & Cuccurullo, 2017).

## Cultural commons in literature: documents analysis

### Scientific production on cultural commons by citations

**Figure 1.**
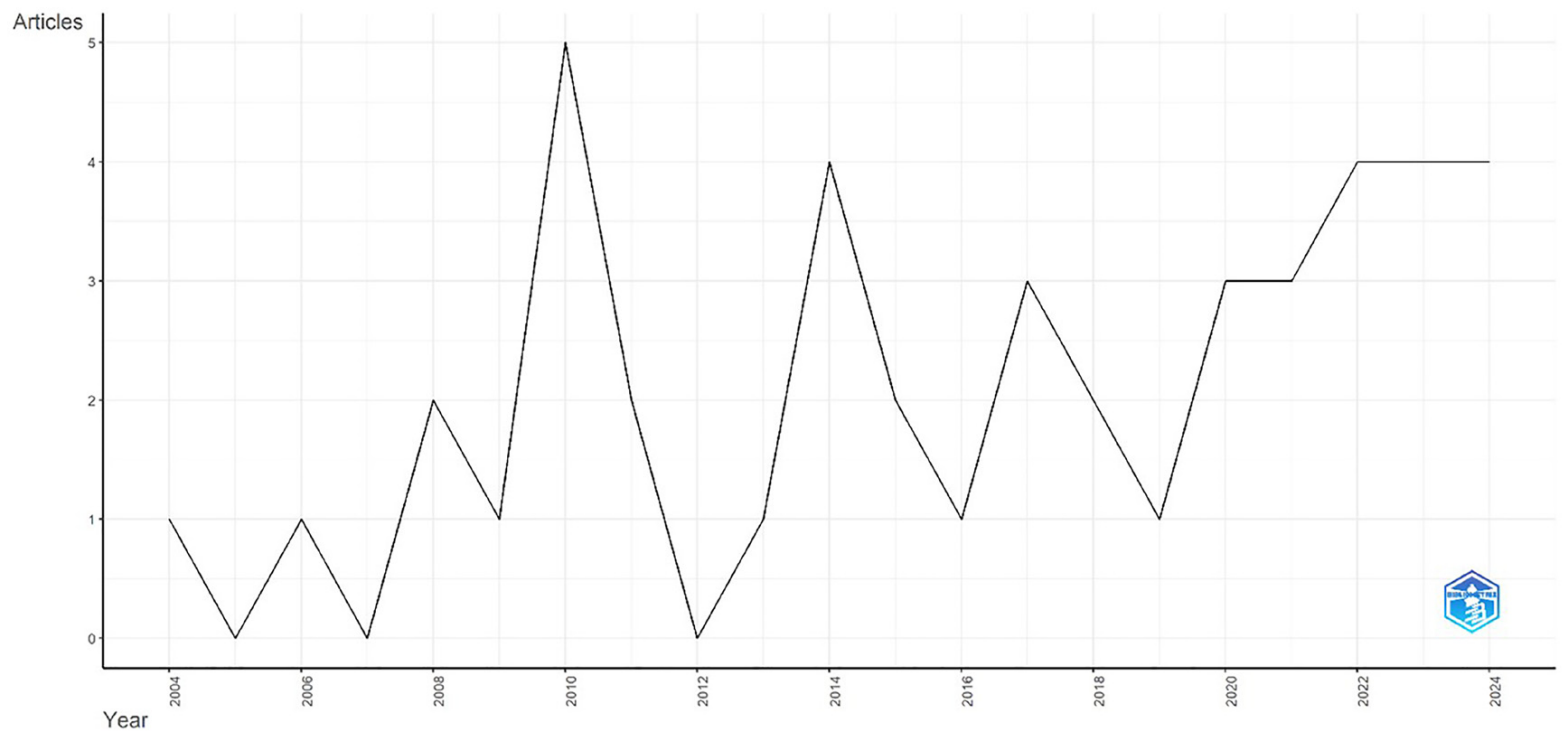
Annual Scientific Production on Cultural commons (2004–2024).

The analysis of the literature on cultural commons shows a positive annual growth rate of 7.18 in the period 2004–2024 ( Figure 1).

As highlighted in Table 1, the analysis of the “Most Global Cited Documents” provides insights into the influence of each work within the broader literature. Specifically, we examined Total Citations (TC) and Total Citations per Year (TC per Year). While TC indicates the overall impact of a publication within its field, TC per Year demonstrates how consistently each document has shaped scholarly discourse since its release.

**Table 1.** Analysis of the Most Globally Cited Documents in the Dataset.

Documents	TC	TC per Year
MADISON MJ, 2010, CORNELL LAW REV	147	9,19
RUTTE C, 2011, BIOL CONSERV	67	4,47
RUTTAN LM, 2008, WORLD DEV	59	3,28
GONZALEZ PA, 2014, CULT STUD	46	3,83
ODUMOSU T, 2020, CURR ANTHROPOL	41	6,83
HOLDER JB, 2008, SOC LEG STUD	41	2,28
TERRAS M, 2015, ONLINE INF REV	40	3,64
MURDOCK G, 2004, EUR J COMMUN	26	1,18
FRISCHMANN BM, 2013, J INST ECON	21	1,62
FUCHS C, 2017, TELEMAT INFORM	19	2,11

The analysis shows that Madison *et al.* (2010) emerges as the most influential document, with a high total citation count (TC = 147) and a strong citation rate (TC per Year = 9.19), suggesting that this contribution has served as a lasting reference in the literature. We can argue that the Madison *et al.* article (2010) reached such a peak because they proposed adapting Ostrom’s original framework for commons to the needs of the cultural commons, arguing that cultural goods or resources operate differently from natural ones, which suggests a different governance structure. Rutte (2011) follows with 67 total citations and a TC per Year of 4.47, indicating continued engagement from the academic community. This contribution advances the understanding of sacred landscapes by elucidating the values, institutional structures, and outcomes that underpin their formation and governance.

In contrast, earlier publications such as Murdock (2004) and Holder & Flessas (2008) exhibit more modest citation rates (TC = 26, TC per Year = 1.18 and TC = 41, TC per Year = 2.28, respectively), suggesting that the impact and relevance of these contributions have diminished over time. Meanwhile, more recent works, such as Odumosu (2020), have had a considerable impact (TC = 41, TC per Year = 6.83), highlighting their rapid integration into contemporary scholarship.

Overall, the analysis of these documents aligns with the evolution of publication outputs over time, as shown in Figure 1. In fact, works such as Madison *et al.* (2010) and Rutte (2011) coincide with one of the peaks in literature production in this field; additionally, the overall citation trends appear to mirror the growing scientific output in recent years, indicating that interest in cultural commons-related topics has steadily increased.

### Scientific production on cultural commons by citations

**Figure 2.**
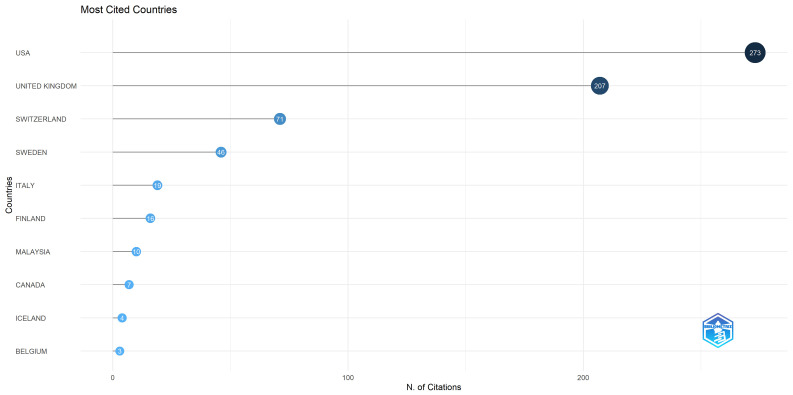
Geographical distribution of citations.

In Figure 2, the “Most Cited Countries” graph extends the analysis by illustrating the geographical distribution of citations. The United States stands out with 373 citations, underscoring its central role in advancing and disseminating research on commons-related topics. The United Kingdom follows with 267 citations, reflecting its robust engagement in this field. This distribution highlights that the Anglo-Saxon world, represented here by the United States and the United Kingdom, appears to be particularly attuned to the discourse on the commons, as both countries have significantly shaped global scholarship in this field. Meanwhile, Switzerland (79 citations), Sweden (71 citations) and Italy (62 citations) underscore Europe’s consistent presence in this domain. Further down the list, Finland (41 citations), Malaysia (35 citations), Canada (27 citations), Iceland (21 citations) and Belgium (19 citations) reveal a significant gap between the top-cited countries and the lower-ranked ones, indicating a citation distribution that is heavily concentrated among a small number of nations.

### Thematic map and gaps in literature on cultural commons

**Figure 3.**
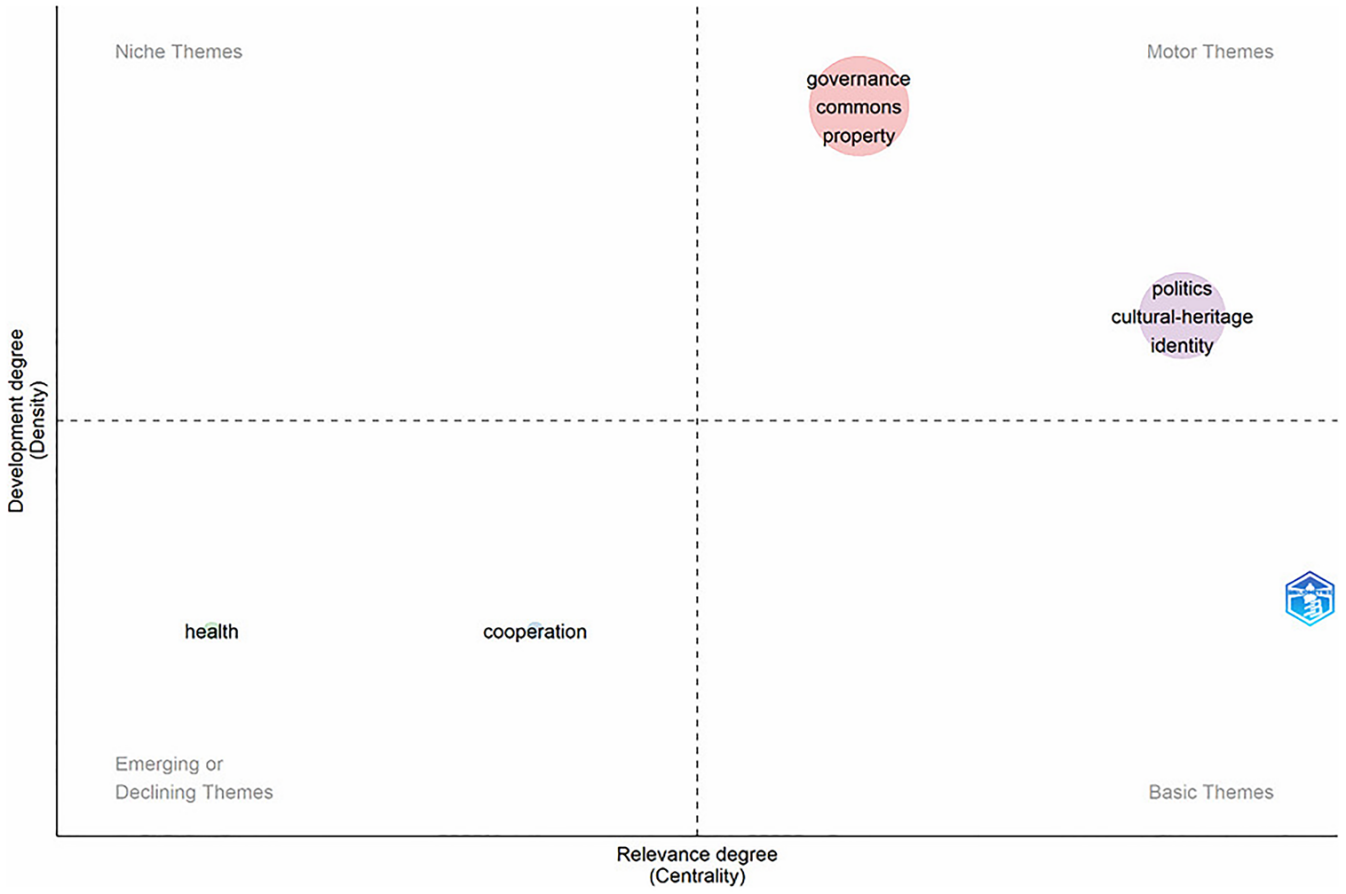
Thematic map of the most developed topics in the literature.

***Content analysis***. The bibliometric thematic map ( Figure 3) highlights the most extensively developed topics in the literature, identifies potential research gaps and pinpoints emerging themes that may offer fruitful avenues for future investigations. The thematic map is a crucial bibliometric tool that organizes the research landscape into clusters based on keyword co-occurrences, co-citation or collaboration network analysis ( Aria & Cuccurullo, 2017), and classifies each theme according to its centrality (i.e. the significance of a theme for the overall research field) and density (i.e. the degree of development or maturity within a specific topic). In this study, the map is constructed using author keyword co-occurrences and reveals three “Motor Themes,” two “Basic Themes”, two “Niche Themes” and four “Emergent or Declining Themes.”

***Motor themes***. The "motor themes" in the quadrant in the upper-right represent clusters that are both highly developed and central to the cultural commons research field. In this quadrant, two interconnected clusters stand out. The first one, including “commons,” “governance” and “property,” emerges with studies highlighting the intricate relationship between commons and property and the challenges of their governance, which remains a central topic in the literature of commons. These findings underscore the need for targeted interventions. The second interconnected cluster combines “politics,” “cultural heritage” and “identity,” which starts to become an intersectional topic in the literature on cultural commons.

***Emerging themes***. The themes in the lower-left quadrant represent emerging topics associated with the core of the research field that appear throughout the selected dataset. It is no surprise, therefore, to find “cooperation” and “health” present within this quadrant. The theme of cooperation is mainly related to the theme of “community,” “conservation” and “forests,” showing how scholars investigate mechanisms and practices as an example of effective management of commons. The second emerging theme is “health,” mainly related to the themes of “community” and “resilience,” which play a significant role in the discourse on commons.

**Figure 4.**
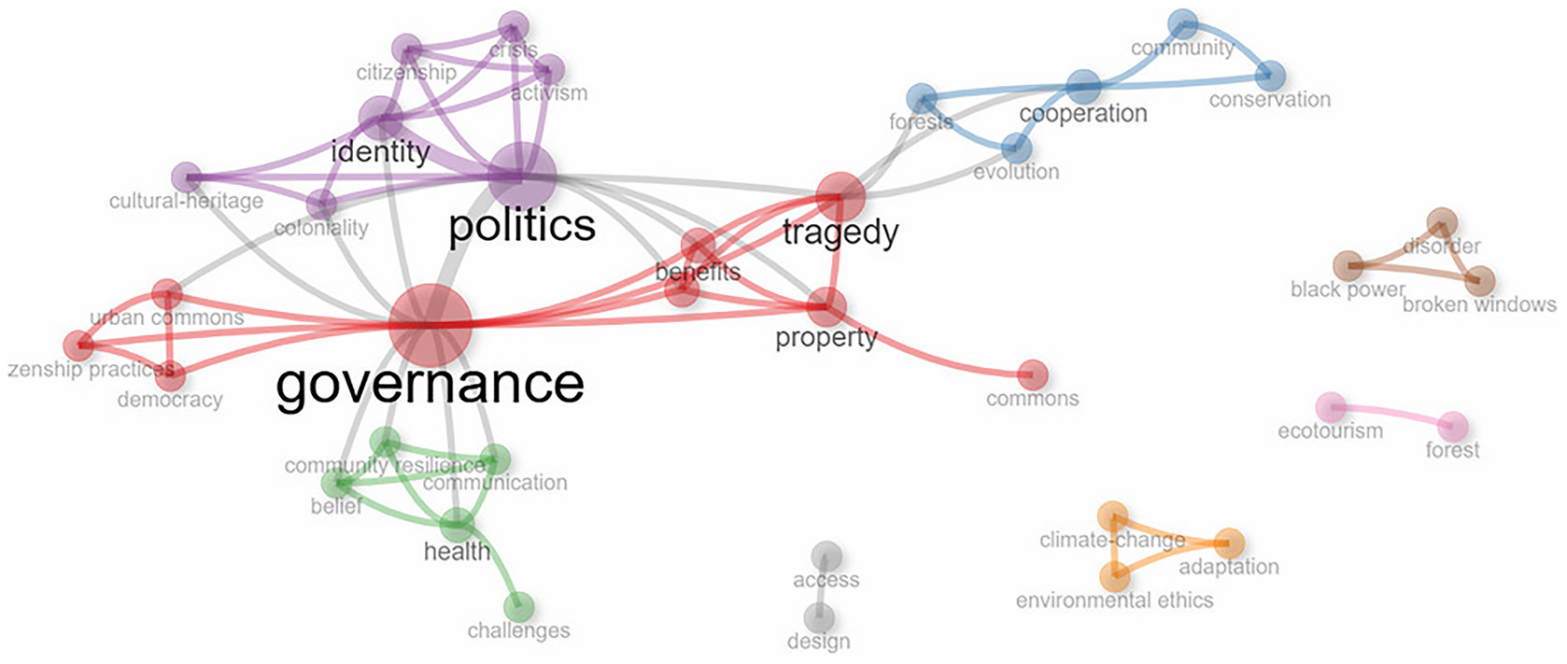
Network of the themes emerged.

Figure 4 presents the networks of the themes emerging in the literature we analysed. As can be seen, three main topics, “governance,” “politics” and “tragedy” stand out. These are themes related to the original stream of literature, starting with Ostrom (1990), and they remain the most evolving themes throughout the literature.

**Figure 5.**
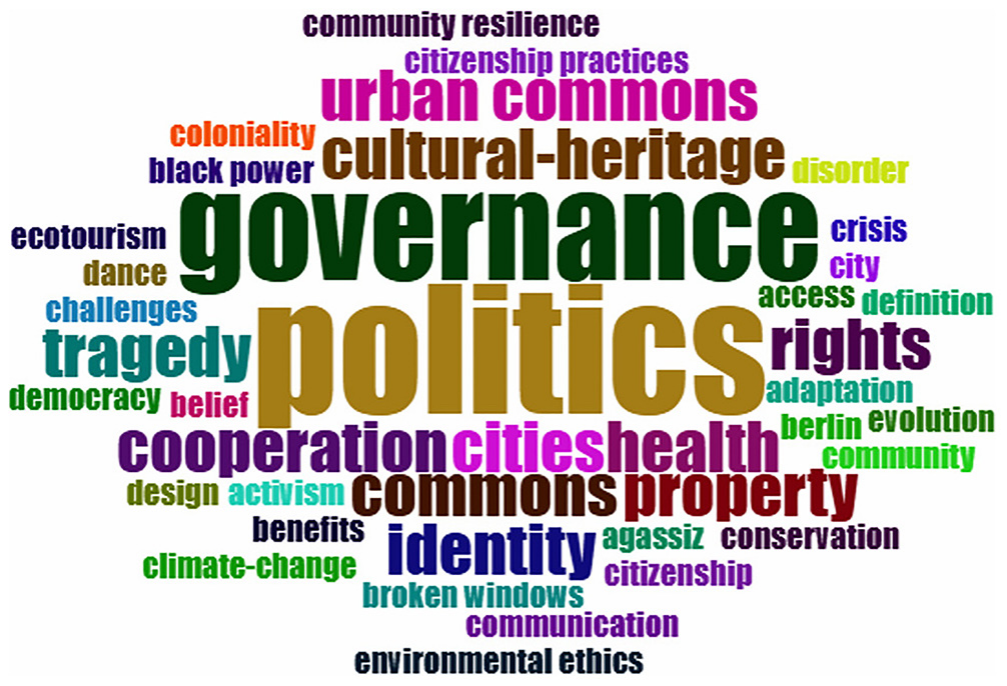
Word cloud of key concepts discussed in the sample of literature on cultural commons.

***Gaps in the research on cultural commons***. We applied the bibliometric analysis of the literature on cultural commons to identify the extent to which the literature reflects the specific characteristics of the cultural goods. Building on the influential work “Constructing Cultural Commons” by Madison *et al.* (2010), we argue that these goods operate or emerge as resources in commons-related contexts with the aim of realising specific cultural, social and societal values. Moreover, they are deliberately constructed systems, as opposed to existing natural resources, and they need to be constructed before being governed ( Madison *et al.*, 2010). Similarly, we argue that studying cultural commons requires not only identifying their structures and governance mechanisms ( Figure 5), but also analysing the underlying values that drive participants to create, maintain and contribute to these commons, i.e. how these values evolve and shape the practices of cultural commons. However, as our discussion of the categories of themes above reveals, only limited academic attention has been paid to analysing an important characteristic of the cultural goods i.e. their values, and the way cultural commons realise them.

## Cultural commons and dynamics of cultural, social and societal values: literature review

Building on the significant contributions of cultural economists, particularly Throsby’s work on valuing cultural goods, and Madison *et al.*'s argument that understanding the values and motivations behind cultural commons is essential for analyzing their governance models, we propose a framework that distinguish the type and nature of cultural resources, whether existing or emerging. This framework also considers the purpose of participation, whether cultural, social or societal, as well as the specific context in which a cultural commons operates.

**Figure 6.**
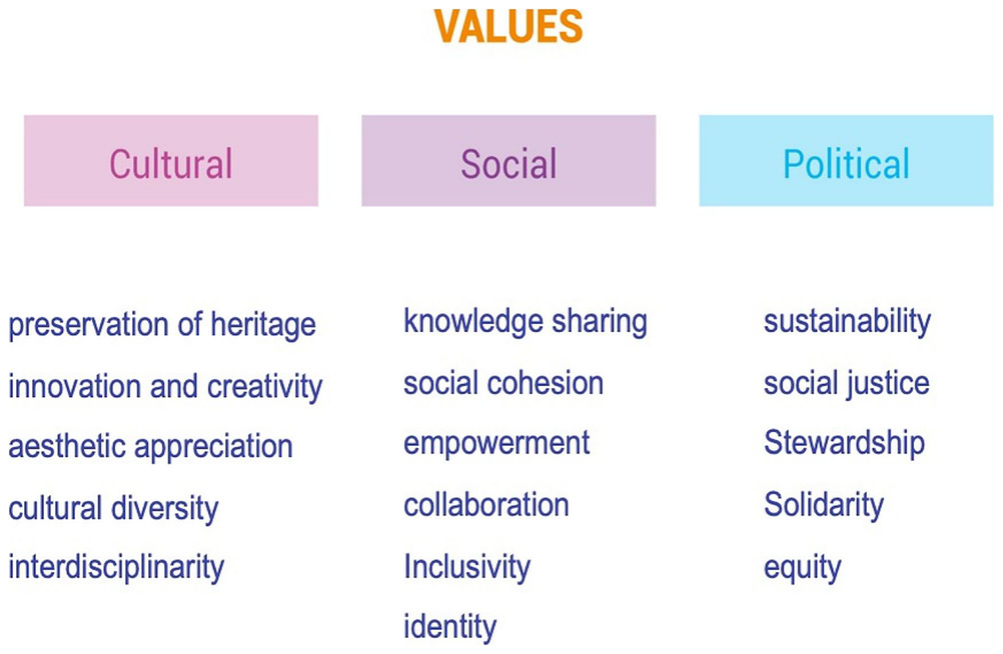
Clusters of values of cultural commons.

The systematic literature review, as well as the previous work of cultural economists, allows us to identify cultural, social and societal clusters of values ( Figure 6) that the cultural commons pursue ( Borchi, 2018; Borchi, 2020; Cominelli, 2014; Fassari, 2020; Gonzalez, 2014; Hess, 2012; Toussaint, 2022; Velkova & Jakobsson, 2017).

These clusters of values differ in terms of their focus, goals and outcomes. For example, whilst cultural values emphasize the preservation, appreciation and innovation of cultural expression, social values instead emphasise community building, inclusivity, collaboration and social justice. Cultural values are aimed at the sustainability and evolution of cultural heritage and the artistic expression of a community, whilst social values seek to foster a cohesive, equitable and participatory community environment ( Petrova *et al.*, 2022). The realisation of cultural values, in terms of outcomes, leads to a rich, diverse and evolving cultural landscape, whereas social values culminate in strong social bonds, empowered individuals and a fair and inclusive community ( Klamer, 2003; Klamer, 2017). Another criterion through which the various cultural commons can be clustered is the nature of the resource *,* i.e. the cultural property or asset (tangible) or practice (intangible).

**Figure 7.**
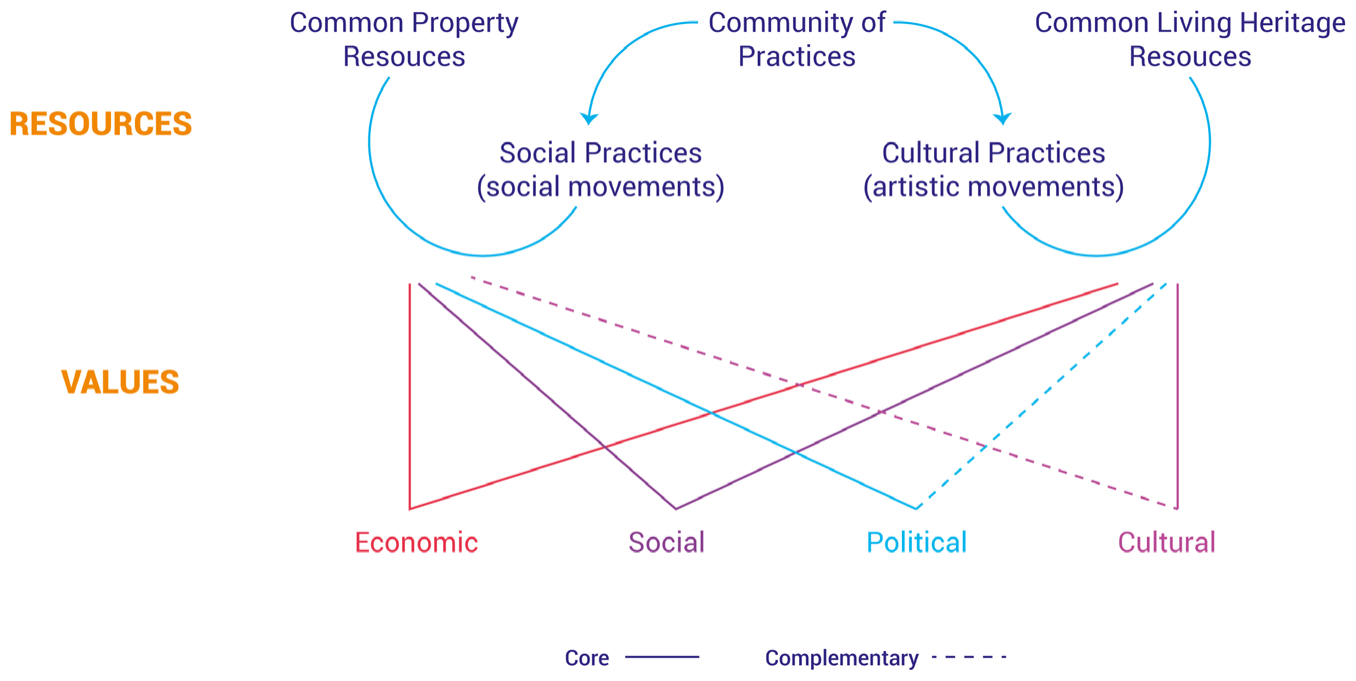
A taxonomy of cultural commons by the nature of the resources and the dynamics between cultural and social value.

Combining both of these criteria, that is, the nature of the resource and its values/purposes, allows us to identify four clusters of cultural commons within the extant literature, which we subsequently organized within the following taxonomy: 1) *commons based on (cultural) property resources,* 2) *community of (cultural or social) practices ^1^* and 3) commons based on *“living heritage” practices ^2^*, ( Figure 7). Although these clusters often intertwine in terms of their resources and purposes, they can nevertheless differ in terms of either their characteristics or the composition of both their participants and outcomes.

Our analysis of the cultural commons based on *common (cultural) property resources* emphasises the importance of community-led collective infrastructures, such as occupied and/or squatted properties, co-working (shared) spaces, abundant spaces appropriated by local communities, landscapes and heritage sites ( Anglada & Santiago Eizaguirre, 2022; Borchi, 2020; Cerreta *et al.*, 2020; Ramos, 2016), or digital platforms ( Clippele, 2023; Dalla Chiesa, 2020; Macey, 2010). Here, the purposes ranged from protesting neoliberal forces, claiming democratic participation as a right to the property/asset and/or the right to work, and building collective multilevel alliances and local autonomous practices. In some cases, the focus lay on the cultural assets of revitalization or re-appropriation. In pursuance of these goals, the commoners practised democratization as a collective learning process and promoted the cultural empowerment of various groups and/or communities via educational and artistic activities. The dynamics between cultural and social values is characterized by an emphasis on social values, which can be enhanced by the specific cultural practice itself (as a process), such as cultural programming. Focusing on the sustainability of the creative work and its cost-effectiveness, these types of cultural practices have a largely supportive function in terms of building a sense of community or identity. Their governance prioritises democratic use and the self-management of shared properties/assets.

*The community of practices* that prioritise *social and/or political values* exhibit characteristics that are similar to the previously discussed types of commons, and can be situated within any of the above listed cultural and collective infrastructures. Their overriding purpose is not to manage property/assets, but rather to enhance social movements. In other words, the focus is not merely on collective property governance, but also on running social experiments and stimulating (social) innovation and citizenship development. More specifically, these centres undertake initiatives that address a large number of the societal challenges we currently face, with the artistic/cultural programming here representing the means through which to address these challenges. In the literature, we identified the following examples within this category: grassroot movements, urban labs, local citizen hubs and existing cultural infrastructures (museum, libraries, etc.) that are open to community events or project-based events with local communities ( Borchi, 2018; De Tullio, 2020).

The cultural commons qua *community of predominantly cultural practices* is often associated with artistic movements, and can be defined as “schools of thought and practice as a common-pool resource (CPR)” or any other intellectual movements (within creative, artistic and scientific fields) ( Fiorentino & Friel, 2012, p.86), the collective production of intellectual property rights and born-digital content ( de Clippele, 2023; Pélissier, 2021; Velkova & Jakobsson, 2017). One distinctive feature of this type, in comparison to the other types of cultural commons, is that their community is led by professionals (although they can also include amateurs and citizens) and often aim to experiment with different cultural (artistic, intellectual) content for the express purpose of contributing towards particular cultural knowledge domains. In this respect, they do not necessarily rely upon strong community/group identity, but rather foster an environment conducive to creative experimentation and innovation by generating a sense of togetherness and/or belonging for the participants. In many instances, these groups are characterized by cross-fertilization between many different disciplines and actors (creatives, scientists, artists, citizens and other professionals) and prioritize, above all, forms of cultural (artistic/intellectual/scientific) innovation and experimentation that are enhanced by social practices (process and outcomes). This is also reflected in forms of shared governance that strive towards facilitating content creation.

The cultural commons based on the *common ‘living heritage’ practices* is associated with the co-curation, co-creation and co-production of traditional cultural knowledge (i.e. intangible resources), for example, members of indigenous or traditional cultural communities ( Cominelli, 2014; Eriksen, 2019). The resources here are intangible in the sense that they consist of folklore and traditional knowledge, such as languages, rituals and so forth, which define the identity of these respective communities. Hence, the purpose here is not so much about building a community identity or a sense of community, but rather about reaffirming a pre-existing identity and sense of community. The embodiment of social and cultural values is thus intertwined much more strongly here than it is in the context of the other types of cultural commons. Social values refer to the shared beliefs and expectations that shape social interactions and relationships. Cultural values, on the other hand, are influenced by multiple determinants, such as language, historical context, traditions, ethnicity and geographical conditions, and provide a useful framework through which to understand what is important and desirable within a given culture, alongside guiding individual behaviour and decision-making. Within this cluster, social values are more closely connected to cultural values but, we must note, they can also vary within different communities. Here, governance is focused on sustaining the traditional knowledge, rituals and practices that serve as the foundation for their daily life.

The cultural commons based on 1) *common (cultural) property resources* and 2a) *community of (social) practices* that prioritise social and/political values operate as part of the social fabric in which cultural activities (cultural programming/co-curation) bring about the realisation of their core social and/or political goals. Conversely, the cultural commons based on 4) *common ‘living heritage’ practices* and *2b) community of cultural practices* are focused on the co-curation and co-creation/co-production of cultural content and/or traditional cultural knowledge.

## Conclusions

When we claim to be using a cultural economics perspective, what we are embracing is a specific approach within the field of cultural economics known as the value-based approach ^3^. This approach, inspired by the work of Throsby, differs markedly from a standard (cultural) economic approach in several key respects. Rather than focusing on transactions amongst individual agents, presuming self-interested agency, and analyzing maximization strategies, a value-based approach emphasises the practices that make up the cultural domain, as well as their social and cultural qualities, and seeks and finds collaborations of all kinds. The latter were found within the co-curation practices we encountered in the analysed case studies. Such practices only occur because people are willing to contribute towards them. By virtue of their contribution, they can be said to develop a sense of common ownership.

Our study focuses on the values that characterise these co-curation practices and on which social dynamics and diverse relationships sustain them. Whilst the commons perspective prioritises the realization of social values, in accordance with the value-based approach we postulate that people engage in shared cultural practices to realise, that is, make real, other values than merely social values, in particular cultural values and financial values. When curators, artists, museum professionals, or other cultural professionals, and scientists, policy makers and members of local communities collaborate, they thus co-create or co-curate new art works, exhibitions, or they experiment with new working methods, and make sense of what the art works signify, all of which involve cultural values. However, they also socialise with people that they otherwise would not meet and, in the process, generate financial revenues.

Cultural values and social values within the cultural commons are intertwined yet distinct concepts, with each contributing towards the overall ethos and functioning of these shared resources. They differ with respect to their focus, goals and outcomes. In essence, cultural values within the cultural commons prioritise the richness and continuity of cultural heritage and artistic expression, whereas social values focus on the well-being, cohesion and equitable participation of the community. Both sets of values are critically important for both the holistic development and sustainability of the cultural commons.

These sets of values reflect different dynamics within the taxonomy of cultural commons that we propose here in this paper. Whilst the cultural commons on *common (cultural) property resources* and *community of (social) practices* prioritise social and/political values, the cultural commons based on *common ‘living heritage’ practices* and *community of cultural practices* underscore cultural values. Acknowledging these differences contributes towards a better understanding of commoning practices, and enriches the perspective that Throsby introduced in cultural economics.

## Ethics and consent

Ethical approval and consent were not required.

## Grant information

This project has received funding from the European Union’s Horizon 2020 research and innovation programme under grant agreement number 101060774.

## Data Availability

The data used ( Petrova *et al.* (2025). What is cultural in cultural commons? https://works.hcommons.org/records/5dfrb-wn242, DOI: 10.17613/5dfrb-wn242 (Data are available under the terms of the Creative Commons Zero "No rights reserved" data waiver (CC0 1.0 Public domain dedication)) in this article is available on this repository: https://works.hcommons.org/records/5dfrb-wn242 DOI: 10.17613/5dfrb-wn242 Data are available under the terms of the Creative Commons Zero "No rights reserved" data waiver (CC0 1.0 Public domain dedication). The project contains the following underlying data: cultural commons file 44 07.02 excel file, size 73Ko. References of publications (articles, book chapters and book). Steps of the research Start: Web of Science (WoS) database >> initial search using the TOPIC (TS) search option. >> following search string: TS=("Cultural common$" OR "Knowledge common$" OR "Digital common$") AND TS=(Cultur*), including truncation operators (“$” and “*”). The search was conducted on January 31 ^st^ 2025 without any publication date restrictions to allow for a broad temporal perspective. Additionally, only documents in English were included in the analysis. First sample identified:144 contributions. We then applied a two-step selection process. First, we conducted a title and abstract screening to exclude contributions considered irrelevant or out of scope, resulting in a refined sample of 32 contributions. Next, to ensure comprehensive coverage, the initial database search was supplemented through manual screening and snowballing techniques, which identified additional relevant articles and journals not captured by the automated query. Analysis of the documents. All retrieved records were subjected to a systematic screening process, in which titles, keywords, and abstracts were reviewed for relevance against the inclusion criteria. The corresponding bibliographic metadata were extracted and stored in a dedicated dataset to facilitate traceability and subsequent analysis. We added 12 further contributions identified as relevant to the bibliometric analysis. They were selected by applying the same key words as described above. This process resulted in a final sample of 44 contributions, which formed the basis of our bibliometric analysis. Finally, to perform the bibliometric analysis presented in the results section, we used Bibliometrix, an open-source package within the R statistical programming environment.

## Footnotes

^1^ Community of practices here refers to “a network …. with shared practices, shared knowledge, identity building and tangible products” ( Feinberg *et al.*, 2023, p. 144).

^2^ Defined by UNESCO (2025, p. 20).

^3^ See Klamer (2017), Klamer *et al.* (2022), Petrova *et al.* (2022).
