# Supplementary material for: What is cultural in cultural commons?
Source: Open Res Eur. 2026 Feb 17;6:52. [Version 1] doi: 10.12688/openreseurope.20908.1 (PMC13329366; doi:10.12688/openreseurope.20908.1)
Supplement: Peer review history

# Reviewer report: Reviewer response for version 1

**DOI:** 10.21956/openreseurope.22619.r75535

**Published:** 2026-07-02

**Authors:** Jen Snowball

**Affiliations:** 1 Rhodes University, Makhanda, South Africa

The paper provides an ambitious and valuable synthesis of several major theoretical traditions in the study of cultural commons. In particular, it successfully brings together Ostrom's seminal work, rooted in environmental economics, with the contributions of Throsby and Klamer within cultural economics. By adopting a value-based approach and employing a quantitative bibliometric analysis of journal articles indexed in the Web of Science database, the article makes an important contribution to the field.

 One of the paper's most significant outputs is the development of an analytical taxonomy/framework for contemporary research on the cultural commons, linked to the categories of value identified through the analysis. This is a highly relevant area of inquiry because, as the authors correctly note, the communal processes of meaning-making and valorisation underpin much of the transactional and financial value generated within the cultural and creative industries (CCIs). The article therefore offers an excellent starting point for scholars seeking to understand both the concept of cultural commons and its relationship to value creation in the CCIs.

 I do, however, have some reservations regarding the conclusion that the United States and the United Kingdom are "particularly attuned to the discourse on the commons, as both countries have significantly shaped global scholarship in this field." While this finding is consistent with the dataset analysed, it may understate the growing body of scholarship emerging from the Global South, particularly research on Indigenous knowledge systems and their relationship to cultural resources governed through long-established and often highly complex institutional arrangements.

 Although the authors provide a convincing justification for their reliance on the Web of Science database, it is important to acknowledge that scholars from the Global South, including those publishing in English, continue to face significant structural barriers in academic publishing, especially within economics and related disciplines. Consequently, the geographical distribution of publications indexed in Web of Science may not fully reflect the global diversity of scholarship on cultural commons. The authors may wish to engage with literature on geographical bias in academic publishing through the given references.

 A further avenue that could enrich the discussion is engagement with the literature on the Solidarity Economy. This body of work foregrounds principles of collective ownership, reciprocity, stewardship, and cooperation, frequently articulated in resistance to the privatization or commodification of common resources. Exploring these connections may provide additional conceptual depth to the analysis of cultural commons and their associated value systems.

 Overall, the paper makes an important and interesting contribution to the literature on cultural commons and provides a valuable taxonomy of different types of cultural goods, linked to the values they create.

If applicable, is the statistical analysis and its interpretation appropriate?

Yes

Is the study design appropriate and is the work technically sound?

Yes

Is the work clearly and accurately presented and does it engage with the current literature?

Yes

Are the conclusions drawn adequately supported by the results?

Yes

Are sufficient details of methods and analysis provided to allow replication by others?

Yes

Are all the source data and materials underlying the results available?

Yes

Reviewer Expertise:

Cultural Economics; Cultural Value and valuation methods; Cultural and Creative Industries.

I confirm that I have read this submission and believe that I have an appropriate level of expertise to confirm that it is of an acceptable scientific standard.

## References

[1] : Who are the Gatekeepers of Economics? Geographic Diversity, Gender Composition, and Interlocking Editorship of Journal Boards. *Review of Political Economy* .2025;37(2) : 10.1080/09538259.2024.2303654 681-708 10.1080/09538259.2024.2303654

[2] : U.S. and them: The Geography of Academic Research. *Journal of Development Economics* .2013;105: 10.1016/j.jdeveco.2013.07.010 112-130 10.1016/j.jdeveco.2013.07.010

# Reviewer report: Reviewer response for version 1

**DOI:** 10.21956/openreseurope.22619.r69979

**Published:** 2026-03-06

**Authors:** Andy C. Pratt

**Affiliations:** 1 City University London, London, England, UK

This paper offers an innovative and timely discussion of the cultural commons and how the disciplinary tools of cultural economics, specifically a 'value based approach' (derived from Throsby), might be helpful.

 The review of the core literatures was well done, and the rational and expansion of the Throsby approach was clear. (although we never really got a clear statement of what a 'value based' approach was (and which values were included and excluded, and how different values could be resolved). The systematic literature review was carried out to clarify the emergent foci of the literatures reviewed.

 In a narrow sense this was logical and clearly done. However, I have a concern that what the SLR achieves is more akin to a taxonomy of objects that are focused on in the literatures: indeed 4 types emerge. On one hand, we could have more elaboration a the implications for the way that cultural economics as a tool is appropriate to each of the objects. On the other hand, a more radical criticism is that cultural economics is in various ways less able to address these objects. 

 This issue of the 'cultural commons' object and its visibility under traditional economic analysis is a key concern. Issues such as organisation, and non-reductive individuation, and of lack of price signals, or reducibility to unitary price or value signals, plus the multi-value value signals (economic, culture , social; critically that - in some readings - are not reducible to economic value. Simply, there is an argument that (unless critically diminished/reduced/simplified) the object of cultural commons is not sensible to cultural economics. A more expansive form of economics, institutional or heterodox that takes multi-valency, non-economic value, plus organisation and power, would seem to be more appropriate to account for the 'lived reality' of the (most versions of) cultural commons.

 Whilst I accept that the paper's viewpoint is valid, I think that some acknowledgement of this alternative  line of criticism and the consequential  de facto limited explanatory power of normative cultural economics to 'explain' /'account for' practices and phenomenon of the cultural commons would be appropriate.

If applicable, is the statistical analysis and its interpretation appropriate?

Yes

Is the study design appropriate and is the work technically sound?

Yes

Is the work clearly and accurately presented and does it engage with the current literature?

Yes

Are the conclusions drawn adequately supported by the results?

Partly

Are sufficient details of methods and analysis provided to allow replication by others?

Yes

Are all the source data and materials underlying the results available?

Yes

Reviewer Expertise:

cultural economics, economic geography, creative industries, institutional economics, cultural studies

I confirm that I have read this submission and believe that I have an appropriate level of expertise to confirm that it is of an acceptable scientific standard.
